# Capturing cognitive and behavioral variability among individuals with Down syndrome: a latent profile analysis

**DOI:** 10.1186/s11689-021-09365-2

**Published:** 2021-04-19

**Authors:** Marie Moore Channell, Laura J. Mattie, Debra R. Hamilton, George T. Capone, E. Mark Mahone, Stephanie L. Sherman, Tracie C. Rosser, Roger H. Reeves, Luther G. Kalb, The Down Syndrome Cognition Project

**Affiliations:** 1 University of Illinois at Urbana-Champaign, 901 S. Sixth St, Champaign, IL 61820 USA; 2 Emory University, 615 Michael St, Atlanta, GA 30322 USA; 3 Kennedy Krieger Institute, 801 N Broadway, Baltimore, MD 21205 USA; 4 Kennedy Krieger Institute, 1750 E. Fairmount Ave, Baltimore, MD 21231 USA; 5 Johns Hopkins School of Medicine, 725 N Washington St, Baltimore, MD 21205 USA; 6 Kennedy Krieger Institute, Johns Hopkins School of Public Health, 3901 Greenspring Avenue, Baltimore, MD 21211 USA

**Keywords:** Down syndrome, Phenotypes, Intellectual disability, Latent profile analysis, Cognition, Adaptive behavior, Maladaptive behavior, Autism symptomatology

## Abstract

**Background:**

There is a high degree of inter- and intra-individual variability observed within the phenotype of Down syndrome. The Down Syndrome Cognition Project was formed to capture this variability by developing a large nationwide database of cognitive, behavioral, health, and genetic information on individuals with Down syndrome, ages 6–25 years. The current study used the Down Syndrome Cognition Project database to characterize cognitive and behavioral variability among individuals with Down syndrome.

**Methods:**

Latent profile analysis was used to identify classes across a sample of 314 participants based on their cognition (IQ and executive functioning), adaptive and maladaptive behavior, and autism spectrum disorder symptomatology. A multivariate multinomial regression model simultaneously examined demographic correlates of class.

**Results:**

Results supported a 3-class model. Each class demonstrated a unique profile across the subdomains of cognition and behavior. The “normative” class was the largest (*n* = 153, 48%) and displayed a relatively consistent profile of cognition and adaptive behavior, with low rates of maladaptive behavior and autism symptomatology. The “cognitive” class (*n* = 109, 35%) displayed low cognitive scores and adaptive behavior and more autism symptomatology, but with low rates of maladaptive behavior. The “behavioral” class, the smallest group (*n* = 52, 17%), demonstrated higher rates of maladaptive behavior and autism symptomatology, but with cognition levels similar to the “normative” class; their adaptive behavior scores fell in between the other two classes. Household income and sex were the only demographic variables to differ among classes.

**Conclusions:**

These findings highlight the importance of subtyping the cognitive and behavioral phenotype among individuals with Down syndrome to identify more homogeneous classes for future intervention and etiologic studies. Results also demonstrate the feasibility of using latent profile analysis to distinguish subtypes in this population. Limitations and future directions are discussed.

**Supplementary Information:**

The online version contains supplementary material available at 10.1186/s11689-021-09365-2.

Down syndrome (DS) is the most common chromosomal disorder among births and is the leading genetic cause of intellectual disability [1]. Individuals with DS have an extra copy of chromosome 21 in all or some of their cells. This genetic difference results in a phenotype that increases the likelihood of certain medical conditions (e.g., congenital heart disease; sleep apnea [2]) and cognitive and behavioral characteristics [3]. These characteristics include lower than average IQ and reduced adaptive functioning, both of which are associated with intellectual disability in general [4]. However, DS also results in phenotype-specific characteristics (e.g., relative difficulties in verbal processing; relative strengths in social functioning [3]) that distinguish it from other neurogenetic disorders associated with intellectual disability. These phenotype-specific features are due to the increased dosage of chromosome 21 genes and resulting dysregulation genome-wide.

Perhaps most striking is the high degree of variability observed among individuals with DS regarding the presence and severity of associated symptoms and conditions. This variability is likely due to a combination of genetic and environmental risk factors that alter the expression of many aspects of the DS phenotype, including cognition, behavior, and health, across the lifespan [5]. However, the nature of this variability in DS is still not well understood, complicating intervention and treatment decisions. We hypothesize that within-syndrome variability can be characterized systematically through the identification of subgroups based on cognitive and behavioral profiles. Thus, the current study takes an individual-differences approach to identify within-group profiles of cognition and behavior among individuals with DS across a wide age range (6–25 years) using latent profile analysis (LPA). The identification of more homogeneous subtypes of individuals with DS may help increase the ability to discover associated genetic and environmental risk factors of severity. Clinically, the resulting data will emphasize different patterns of strengths and areas in need of additional support among individuals with DS. Ultimately, more precision in characterization of the individual variability within DS will lead to more targeted treatment and intervention approaches to optimize daily function for this population. In the current study, we focused on the domains of cognition (IQ and executive functioning), adaptive and maladaptive behavior, and autism spectrum disorder (ASD) symptomatology.

## Challenges to the assessment of the cognitive and behavioral phenotype of DS

### Cognition

Research on cognition in DS has focused broadly on IQ and executive functioning. IQ represents overall cognitive ability and is thought to broadly impact other areas of functioning (e.g., adaptive behavior, maladaptive behavior). Another particularly important area of cognition is executive functioning, which encompasses the higher-order cognitive and self-regulatory abilities that are important for goal-directed behavior and daily functioning (e.g., social interactions, academic achievement, work behavior [6–10]). The abilities housed under the umbrella term of executive functioning include working memory, inhibition, shifting/cognitive flexibility, attention, and planning. Both IQ and executive functioning are important components of the DS phenotype, while also showing inter-individual variability [11].

#### IQ

IQ is perhaps one of the more variable constructs among individuals with DS, ranging from approximately 40 (or the lowest score possible on many standardized intelligence tests) to around 70 (the cutoff score for intellectual disability) and in some cases even higher [12]. There are two main issues associated with interpreting IQ scores in individuals with DS: use of age-based norms and scoring at the floor (i.e., the lowest score possible [13, 14]).

Like others with intellectual disability, the rate of growth in cognitive skills is slower for individuals with DS than in the general population. Therefore, the use of age-based norms (i.e., IQ standard scores) from the general population leads to a discrepancy that becomes larger with age. This discrepancy causes IQ standard scores to decrease, or at least not increase, over time, despite continued growth in cognitive skills [12, 15–17].

Using age-based norms can also lead to many individuals with DS and intellectual disability scoring at the floor. To address this problem, some research groups have developed *Z*-score-based deviation IQ scores that account for performance below the floor-level standard score (Hessl et al. [18] for the Wechsler Intelligence Scale for Children-III; Sansone et al. [13] for the Stanford Binet-5). Another solution to the floor effect problem is to use raw scores or growth scores that represent performance on a standardized IQ test without taking into account age-based norms [16]. This approach is especially useful for interpreting growth over time. For example, a review of longitudinal studies using the Stanford Binet IV to assess individuals with DS from age 4 to 24 years found that 37% scored at the floor level, and thus, growth could not be examined with standard scores [12]. Raw scores, however, increased over time. The rate of growth over time, measured using age equivalent scores, was non-linear and highly variable across individuals. Interpretation of these results is limited due to their use of age equivalent scores which do not provide accurate estimates of ability level and have poor psychometric properties [19]. However, these results do highlight the high degree of variation associated with the DS phenotype. The current study takes into account this variation by examining age-corrected raw scores from another IQ measure, the Kaufman Brief Intelligence Test, 2nd edition (KBIT-2 [20]), in the profile analyses.

#### Executive functioning

Executive functioning is an area in which many individuals with DS struggle, compared to both chronological and mental age-matched peers [3, 21]. Within executive functioning, the areas of working memory, planning, and inhibition are particularly difficult for individuals with DS; emotional control is considered a relative strength [22, 23]. Although this pattern appears to be relatively consistent across development, Loveall et al. [23] noted some nuanced differences in the executive functioning profile in childhood and adulthood.

Working memory is the component that has been examined in greater depth across the literature on DS than other abilities under the executive functioning umbrella. Defined as the simultaneous storing and manipulation/processing of information [24, 25], working memory often has been characterized as an area of weakness for individuals with DS, with greater difficulties reported in verbal working memory than visuospatial working memory [26, 27]. Verbal working memory appears to be weak compared to both mental age-matched typically developing children [28, 29] and individuals with other intellectual and developmental disabilities [30–33]; it also develops at a slower rate in individuals with DS than in peers with other intellectual and developmental disabilities [34].

Although historically, visuospatial working memory has been thought to be intact in the DS phenotype [26], recent research suggests variability in performance among individuals with DS across different visuospatial working memory tasks [35]. Collectively, the literature points to impairments in the spatial-simultaneous [36–38] and visual [39, 40] components, with the spatial-sequential component relatively intact [41].

### Adaptive behavior

Adaptive behavior involves the skills needed to function independently in everyday life, such as self-care, domestic function, and community interactions [4, 42, 43]. These skills are critical to positive long-term outcomes, including academic success and independence [44–47]. Research on adaptive behavior generally categorizes associated skills into four broad domains: socialization, communication, daily living, and motor skills.

Individuals with DS demonstrate relative strengths in socialization and difficulties in communication and motor skills [48–51]. As for daily living, the research is mixed on whether this is an area of strength or difficulty [47, 49]. However, Daunhauer [47] analyzed within-group effect sizes for previously conducted adaptive behavior studies and noted that daily living skills may actually be an area of relative difficulty due to its overlap with other adaptive domains. For example, being able to dress oneself is a daily living skill, but this can be difficult if an individual has compromised motor skills. Regardless, adaptive behavior is holistically an area of difficulty for individuals with DS, again with considerable inter-individual variability.

One of the challenges to understanding adaptive behavior within the DS phenotype is that it is often examined separately from other aspects of the phenotype. However, it is important to recognize that one’s ability to function independently is likely intertwined with other aspects of cognition and behavior. For example, both executive functioning and IQ are strongly associated with level of adaptive functioning [21, 22] and may be related to the presence of maladaptive behaviors [47, 52]. The present study is specifically designed to investigate within-group profiles of adaptive behavior relative to IQ and executive functioning, rather than simply comparing sample means across these constructs.

### Maladaptive behaviors

In contrast to adaptive behavior, maladaptive behaviors represent a range of behaviors that impede health, well-being, educational and occupational outcomes, and daily functioning. These behaviors are traditionally categorized as internalizing (e.g., anxious or depressed symptoms) or externalizing (e.g., inattention/hyperactivity; aggressive behaviors). Higher levels of maladaptive behaviors have been noted in individuals with DS who also have identified comorbid neurobehavioral disorders (e.g., ASD; disruptive behavior disorder [53–56]).

Children and adolescents with DS have higher rates of maladaptive behaviors than the general population [57–59]. However, the within-DS profile of maladaptive behaviors appears nuanced. For example, population-based studies of Dutch individuals with DS showed high rates of problems in the “social” and “inattention” domains on the Child Behavior Checklist, but relatively low rates of internalizing problems in the “anxious-depressed” domain [58, 59]. Although scores in the “delinquent behavior” and “aggressive behavior” domains were slightly elevated in the child cohort, the effect size was small, suggesting a nuanced difference at best [58]. A developmental pattern also emerged such that more difficulties with “anxious-depressed” symptoms were found in the adolescent group than in younger children. This pattern is consistent with other within-syndrome reports as well as the broader literature on internalizing behaviors in adolescents [57, 60]. One aspect that remains unclear is how maladaptive behaviors relate to cognition. The current study directly addresses this gap by using cluster profiles to examine these associations.

### Comorbid autism spectrum disorder (ASD) in DS

Individuals with DS are at a greater risk of comorbid ASD than the general population, with prevalence estimates suggesting up to 19% [61]. Notably, comorbid ASD tends to impact both cognitive and behavioral aspects of the DS phenotype. Compared to individuals with DS who do not have comorbid ASD, individuals with DS and comorbid ASD tend to display more maladaptive behaviors (i.e., stereotypic and anxious/withdrawn domains [53, 55]) and lower IQ and adaptive functioning [61, 62]. Additionally, studies examining broader ASD-like symptomatology among individuals with DS who are at *low risk* for comorbid ASD have found a similar pattern; lower IQ and adaptive functioning and higher rates of maladaptive behaviors are associated with broader ASD-like symptoms [63–65]. Therefore, it appears that there is some overlap between ASD-like symptoms and the behavioral phenotype associated with DS. However, it is unclear if the use of group-level means obscures a more nuanced profile of cognition and adaptive behavior in relation to ASD symptomatology in DS, similar to what is observed in non-syndromic ASD [66].

## Current study

The literature on cognition (IQ and executive functioning), adaptive and maladaptive behaviors, and ASD symptomatology associated with the DS phenotype indicates a general syndrome-specific pattern; however, as reviewed above, it also indicates significant variability. Thus, we hypothesize that the general syndrome-specific pattern does not represent all individuals with DS and that within-group profiles can be identified. More nuanced within-group profiles may vary by age because general developmental differences within the syndrome-specific pattern of strengths and weakness have been noted in the literature. Thus, we also hypothesize that age is associated with within-group profiles.

Using LPA as an exploratory approach, we aimed to test our hypotheses by (1) determining whether subgroups of individuals with DS can be characterized using performance-based and caregiver-informed ratings of behavior and cognition and (2) examining the demographic characteristics associated with the latent subgroups. To achieve these aims, we studied a large sample of individuals with DS, ages 6–25 years, who were recruited through a network of sites across the USA called the Down Syndrome Cognition Project (see Rosser et al. [14]).

## Methods

### Participants

Three hundred fifty-six individuals with DS, ages 6–25 years old, participated in the larger study, the Down Syndrome Cognition Project (see Rosser et al. [14] for inclusion criteria and participation rates). Using that sample, we retained all members of the original cohort except for participants who were missing data on three or more of the primary measures (*n* = 42), resulting in a final sample size of 314. Participants were recruited from clinics, community events/support groups, conferences, advertisements, and previous research studies by multiple sites across the USA. See Table 1 for participant demographic information.

**Table 1:** Demographic characteristics of the overall sample and latent classes

	Overall Sample	“Normative” class	“Cognitive” class	“Behavioral” class
*N* (%)	314 (100)	153 (48.7)	109 (34.7)	52 (16.6)
Age (*M*, *SD*)	13.7 (4.7)	13.6 (4.5)	13.5 (4.7)	14.6 (5.1)
Maternal age (*M*, *SD*)	34.8 (5.5)	35.4 (5.3)	34.3 (6.2)	34.2 (4.5)
Paternal age (*M*, *SD*)	36.4 (5.7)	37.0 (5.5)	35.7 (6.1)	35.9 (5.3)
Household income (%)				
<$75,000	28.7	22.3	34.9	31.4
$75,000–$100,000	15.5	14.4	17.0	15.7
$100,000+	55.8	62.3	48.1	52.9
Sex (% male)	51.0	56.9	40.4	55.8
Self-identified race/ethnicity (%)				
White	75.2	80.8	67.6	75.0
Black	6.4	6.6	8.3	1.9
Hispanic	3.2	1.3	6.5	1.9
Asian	14.5	11.3	16.7	19.2
Other	.6	0	1	1.9
Congenital heart defect (% yes)	61.0	65.6	54.4	60.8
Assessment scores (*M*, *SD*)				
KBIT-2	25.2 (11.7)	30.0 (10.8)	16.3 (8.5)	29.9 (9.0)
CANTAB spatial span	2.7 (1.6)	3.3 (1.2)	1.4 (1.6)	2.9 (1.4)
NEPSY-II visuomotor precision	13.3 (5.7)	14.2 (5.6)	11.5 (6.0)	14.4 (5.1)
BRIEF	135 (23.3)	122.7 (19.3)	144.2 (19.5)	154.3 (20.2)
SIB-R	57.9 (23.3)	70.4 (19.6)	39.3 (18.2)	58.3 (16.7)
NCBRF conduct problems	7.5 (6.1)	5.1 (4.1)	7.5 (5.3)	14.8 (6.6)
NCBRF insecure/anxious	4.0 (4.0)	2.7 (2.5)	2.9 (2.5)	10.0 (4.7)
NCBRF hyperactivity	6.5 (4.4)	4.5 (3.2)	7.7 (4.2)	10.1 (4.7)
SCQ	8.6 (5.5)	5.2 (3.2)	12.5 (4.9)	11.8 (5.5)

*KBIT-2* Kaufman Brief Intelligence Test, 2nd edition, *CANTAB* Cambridge Neuropsychological Test Automated Battery, *BRIEF GEC* Behavioral Rating Inventory of Executive Function, *SIB-R* Scales of Independent Behavior-Revised, *NCBRF* Nisonger Child Behavior Rating Form, *SCQ* Social Communication Questionnaire

### Measures and procedure

The Down Syndrome Cognition Project testing battery [14] included a series of performance-based neuropsychological assessments modified from the Arizona Cognitive Test Battery, which was developed to produce outcome measures for clinical trials involving individuals with DS [67, 68]. The test battery has good test-retest reliability [67].

Examiners at each Down Syndrome Cognition Project site were trained to fidelity to administer the testing battery to individuals with DS (see Edgin et al. [67, 68]). Sites were monitored by the University of Arizona training site and/or the Emory Data Coordinating Center. Periodic quality control checks included review of fidelity to administration and scoring procedures. All data were double entered with comparison checks to ensure accuracy. Research Electronic Data Capture (REDCap [69]) was used for data entry and management.

Participants completed the testing battery at an assessment site or another convenient location (e.g., community center or family home). The entire session lasted about 2–3 h with frequent breaks. Tests were presented in two fixed, counterbalanced orders [67]. Caregivers completed questionnaires related to participant demographics, medical/developmental history, and behaviors.

Table 2 includes a description of the primary measures included in the model. These tests were selected based on several criteria. Most importantly, each measure was selected on the basis of reflecting an important and unique component of cognition, behavior, or development. Where possible, we used total composite or summary scores from the indicated measure to capture all salient information and reduce the number of overall variables. Variables were also selected based on normality. Variables that were highly skewed, due to floor or ceiling effects, limited the ability to measure the full range of abilities and discriminate among subgroups, and thus were eliminated from the analyses. Finally, measures were also selected based on number of observations, because not all measures were administered to the entire cohort.

**Table 2:** Description of primary measures

Domain of assessment	Measure	Outcome variable^a^	Interpretation of score
*Performance-based assessments of cognition*			
Verbal + nonverbal intelligence	KBIT-2	Raw score composite	Higher score = more skills
Working memory (immediate memory for spatial-temporal sequences)	CANTAB Spatial Span	Forward span length	Higher score = more skills
Visuomotor scanning and hand-eye coordination	NEPSY-II Visuomotor Precision, ages 3–4	Total score (time and number of errors)	Higher score = more skills
*Caregiver-informed assessments of cognition and behavior*			
Executive functioning	BRIEF School Age	Global Executive Composite standard score	Higher score = more impairments
Adaptive	SIB-R Short Form	Standard score composite	Higher score = more adaptive skills
Maladaptive	NCBRF	Conduct problems, insecure/anxious, and hyperactivity subscale raw scores	Higher score = more maladaptive behaviors
Autism spectrum disorder (ASD) symptomatology	SCQ-Lifetime	Total raw score	Higher score = more ASD-like symptoms

*KBIT-2* Kaufman Brief Intelligence Test, 2nd edition, *CANTAB* Cambridge Neuropsychological Test Automated Battery, *BRIEF* Behavioral Rating Inventory of Executive Function, *SIB-R* Scales of Independent Behavior-Revised, *NCBRF* Nisonger Child Behavior Rating Form, *SCQ* Social Communication Questionnaire

^a^All outcome variables were age-corrected

#### Performance-based assessments of cognition

Three performance-based tests of cognition were included in this study. Intelligence was evaluated using the Kaufman Brief Intelligence Test, 2nd edition (KBIT-2 [20]). Verbal and nonverbal raw scores were summed and then averaged for use in the model. This approach was taken because the IQ composite variable had significant floor effects (34% of the sample had an IQ ≤40). Working memory was evaluated using the Cambridge Neuropsychological Test Automated Battery (CANTAB) Spatial Span test, which required the participant to recall progressively longer sequences of visual information (colored squares) presented on a touchscreen. Visuomotor tracking and hand-eye coordination were evaluated using the NEPSY-II Visuomotor Precision Task (ages 3–4). For this task, the participant used their preferred hand to draw a line as quickly as possible while staying inside the tracked lines [70].

#### Caregiver-informed measures

Four caregiver-report measures of cognition and behavior were included. Executive functioning impairments were assessed via the Global Executive Composite standard score from the Behavioral Rating Inventory of Executive Function, School Age (BRIEF [71]). Adaptive behavior was evaluated using the standard composite score of the Scales of Independent Behavior-Revised, Short Form (SIB-R [72]). Maladaptive behavior was assessed across three variables (conduct problems, anxiety, and hyperactivity) from the Nisonger Child Behavior Rating Form (NCBRF [73]). These subscales were selected because the additional subscales overlapped with the other measures employed in this study (e.g., ASD symptoms, adaptive behavior). ASD symptoms were assessed via the Social Communication Questionnaire, Lifetime version (SCQ [74]). The total raw score from the SCQ was used. All measures described above are psychometrically sound assessments used frequently in individuals with DS. See Edgin et al. [67] for more detailed information.

Demographic information on participant age, sex, self-identified race and ethnicity, presence of a congenital heart defect, household income, and maternal and paternal age were obtained via caregiver report and medical record abstraction (Table 1).

### Statistical analysis

**Fig. 1:**
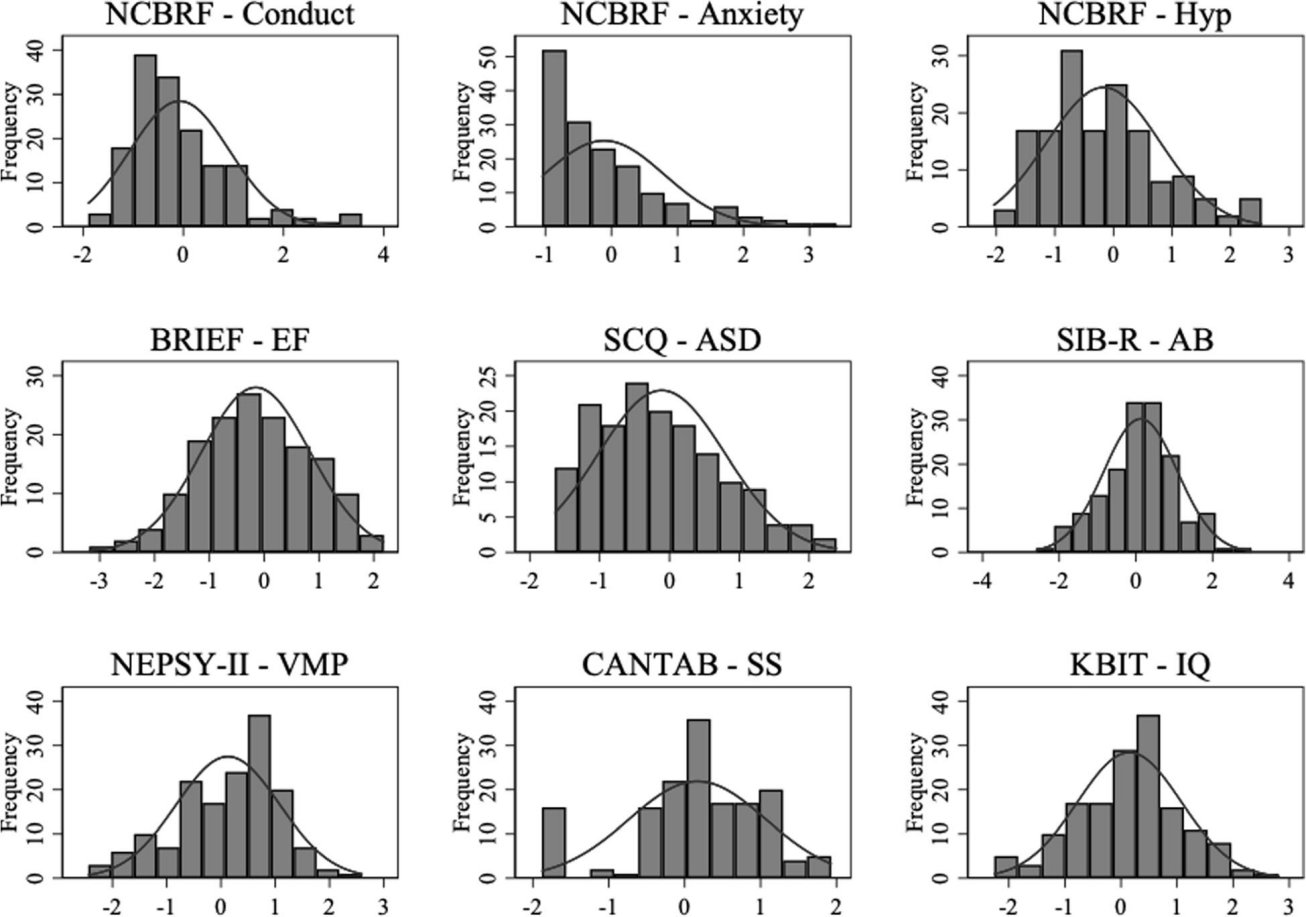
Distribution of age-corrected, Z-score transformed measures used in the latent profile analysis. *Note.* NCBRF Nisonger Child Behavior Rating Form, Hyp hyperactivity, BRIEF Behavioral Rating Inventory of Executive Function, *EF* executive functioning, SCQ Social Communication Questionnaire-Lifetime, *ASD* autism spectrum disorder, *SIB-R* Scales of Independent Behavior-Revised, *AB* adaptive behavior, VMP visuomotor precision, CANTAB Cambridge Neuropsychological Test Automated Battery, SS spatial span, KBIT Kaufman Brief Intelligence Test, 2nd edition, IQ verbal and nonverbal raw score sum (averaged)

The first step in the analysis was to place all of the variables on a common metric, because many of the variables were based on raw scores. If items did not have similar scaling, the LPA would be uninterpretable. To accomplish this, all variables were transformed using a *Z*-score conversion, which placed all scores on a metric of Mean = 0 (reflecting the overall sample average) and SD = 1. Thus, *Z*-score values can be interpreted in terms of standard deviations. For instance, a 1-point SD difference is equivalent to a 68.2% difference between groups. This is interpreted as a large effect size, whereas a 0.5 SD (34.1% difference) represents a moderate effect size. During the transformation, all variables, including standard scores, were adjusted for age. All variables retained their original direction of measurement, such that higher scores on the NCBRF, SCQ, and BRIEF indicated greater impairment, and higher scores on the performance-based measures and SIB-R reflected less impairment/stronger performance. See Fig. 1 for visual representation of each age-corrected, *Z*-score transformed variable.

Next, the age-corrected, *Z*-score transformed variables were submitted to an LPA. LPA was used to identify homogeneous, latent subgroups of individuals with DS based on continuous indicators of cognition, behavior, and development [75]. Using a series of fit statistics, the first step in this procedure is to identify how many subgroups (hereafter referred to as “classes”) existed in the sample. The most commonly known fit statistics used in this study were the Akaike (AIC) and sample size-adjusted Bayesian (SS-BIC) information criterions. Lower AIC and SS-BIC values indicate a model that fits the data best [76]. Next, Vuong-Lo-Mendell-Rubin (VLMR) and bootstrapped likelihood ratio (BLRT) tests were employed. These tests iteratively examined the current model compared to the previous model with one less class. When *p* > .05, it suggests that the current model does not fit significantly better than the *K*-1 model [75]. Additional metrics included entropy and mean class probability (M[CP]), both of which indicate misclassification. Higher scores on both metrics are best, with a value of 1 being perfect measurement. Prior work by Nylund et al. [77] suggests that the BIC and BLRT are more reliable indices of class structure than the AIC and VLMR, respectively.

After arriving at the optimal class structure, individual membership to the model was exported. Each individual was assigned to a class by the software based on the class that represented their greatest posterior probability of membership. Finally, a multivariate multinomial regression model simultaneously examined all demographic correlates of class. Only paternal age was excluded because it was strongly associated with maternal age (*r* = .71). Self-identified race/ethnicity was dichotomized (White vs. other) because there were small sample sizes for non-White categories. The multinomial model included robust variance to address any unknown clustering or model misspecification. LPA analyses were performed in MPLUS Version 7.0 [78], and all other data management and analysis occurred in STATA 15.0 [79].

Three variables used in the LPA measurement model had substantial missingness (SIB-R, 7% missing; CANTAB, 21%; SCQ, 23%). MPLUS uses full information maximum likelihood estimation (FIML) to accommodate missing data in structural equation-based models. FIML produces unbiased estimates and standard errors when data are missing either at random or completely at random [80]. To understand the influence of FIML, the LPA was run on the sample using complete case analysis, which severely limited the sample size (*n* = 156). Importantly, the findings supported the same inferences as the model that included the entire sample via FIML in terms of class structure and fit statistics. Given this finding and the fact that complete case analysis has its known biases, the full sample was employed in the LPA and regression analyses using FIML.

## Results

### LPA

Fit statistics for the iterative LPA procedure are shown in Table 3. Determining the number of classes to include in the model depends on assessing the various fit statistics as well as considering class size and interpretation. We examined 2- through 4-class models. Table 3 shows that the AIC and SS-BIC values decreased continuously with increasing class size, indicating a better fit with increasing number of classes, as expected. The decrease in these scores was considerably larger between the 2- and 3-class models compared to the 3- to 4-class models. This pattern provides evidence of at least a 3-class model. Entropy and M(CP) were high and fairly consistent across the models. The VLMR suggested a 2-class model, as the 3-class model did not significantly improve the model fit. However, the BLRT remained significant at *p* < .001 with the 4-class model, suggesting more than four classes may be indicated. A fifth class was not evaluated due to the small class size in the 4-class model.

**Table 3:** Fit statistics from the latent profile analysis

Number of classes	AIC	SS-BIC	Entropy	M(CP)	VLMR	BLRT	Smallest Class Size
2	7172	7188	.76	.92	<.01	<.001	39
3	7024	7046	.80	.90	.15	<.001	18
4	6968	6996	.80	.89	.32	<.001	7

*AIC* Akaike information criterion, *SS-BIC* sample size-adjusted Bayesian information criterion, *M(CP)* mean class probability, *VLMR* Vuong-Lo-Mendell-Rubin Test *p* value, *BLRT* bootstrapped likelihood ratio test *p* value

Overall, three indices (AIC, BIC, BLRT) provided evidence for more than two classes, whereas the VLMR suggested a 2-class solution. Prior work by Nylund et. al [77] has shown BIC and BLRT as two of the most reliable fit indices, both of which supported a larger number of classes in this dataset. Given that the fit statistics generally supported more than two classes, and the 2-class solution did not reflect important heterogeneity detected in the 3- and 4-class solutions, the 2-class model was not selected.

The 4-class model is shown in Figure 1 of the supplemental information. The fourth class demonstrated the greatest impairments, both cognitively and behaviorally. Notably, the sample size was small (7%) for this class. The small sample size raises concerns about reproducibility as well as diminished power when examining demographic differences. As such, the 3-class model was chosen.

**Fig. 2:**
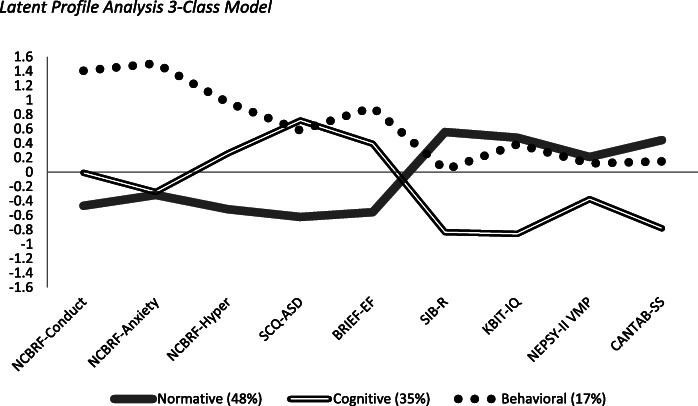
Latent profile analysis 3-class model. *Note.* All values are *Z*-score transformed (*M* = 0; *SD* = 1). Higher scores on the NCBRF, SCQ, and BRIEF indicate greater impairment; higher scores on all other measures reflect less impairment

Figure 2 displays the 3-class model that we adopted and is consistent with our hypothesis that subtypes can be characterized based on the variability observed in cognitive and behavioral measures. We termed the classes based on the variables around which each group clustered rather than describing the participants themselves. The largest group (48%) had an even profile with respect to all domains included in the analysis (Fig. 2, gray line); we termed this the “normative” class. This class was demarcated by relatively strong performance compared to the sample average. That is, negative values indicated less maladaptive behaviors, ASD symptomatology, and executive functioning impairments. Positive scores on the SIB-R and all performance-based measures indicated stronger cognitive skills (IQ and visuospatial abilities) and adaptive behavior compared to the sample average. The second largest group (double line) was termed the “cognitive” class (35%). This class was distinguished by low scores on the performance-based measures of cognition; however, measures of ASD symptomatology, executive functioning impairment, and hyperactivity were also elevated, indicating difficulties in these areas. The final group (dotted line) was termed the “behavioral” class (17%). This class had highly elevated scores for maladaptive behaviors, followed by elevated ASD symptomatology and executive functioning impairments. Conversely, their cognitive abilities from the performance-based assessments and adaptive behavior were all near or above the sample average, suggesting these abilities were similar to the “normative” group.

### Demographic correlates

A multivariate multinomial regression model was used to simultaneously examine all demographic correlates of class. The “normative” class served as the reference group. Only two variables were significantly different across the latent classes (Table 4). Participants in the “cognitive” class were 47% less likely to have a family income of > $100,000 and 49% less likely to be female when compared to the “normative” class (both *p* < .05). No differences were found among the demographic variables between the “normative” and “behavioral” classes. See Table 4 for details.

**Table 4:** Results from the multivariate multinomial regression model

Demographic variable	“Cognitive” class		“Behavioral” class	
	RRR	95% CI	RRR	95% CI
Age	.98	.92, 1.05	1.04	.97, 1.12
Maternal age	.97	.92, 1.02	.97	.92, 1.02
Income				
<$75,000	Ref	Ref	Ref	Ref
$75,000–$100,000	.75	.32, 1.75	.89	.32, 2.50
$100,000+	.53*	.29, .84	.69	.21, 1.48
Sex				
Male	Ref	Ref	Ref	Ref
Female	.50*	.29, .83	.98	.51, 1.89
Self-identified ethnicity				
White	Ref	Ref	Ref	Ref
Non-White	.61	.32, 1.14	.68	.31, 1.50
CHD				
No	Ref	Ref	Ref	Ref
Yes	.67	.39, 1.14	.82	.42, 1.62

*Ref* reference group (“Normative” class serves as the overall reference group), *CHD* congenital heart defect, *RRR* relative risk ratio

*p <.05

## Discussion

The current study examined variability in the DS phenotype among 314 children, adolescents, and young adults with DS, a large sample size for this population. Specifically, we aimed to (1) determine whether subgroups of individuals with DS could be characterized using performance-based and caregiver-informed ratings of behavior and cognition and (2) examine the demographic characteristics associated with the latent subgroups. Using LPA, we identified three distinct classes of individuals with DS, referred to as the “normative,” “cognitive,” and “behavioral” classes; these labels are based on the variables around which the three groups clustered rather than the groups themselves.

Representing almost half of the sample (48%), the “normative” class demonstrated a relatively consistent profile with respect to cognition and adaptive behavior, including relatively low levels of maladaptive behavior, ASD symptomatology, and executive functioning impairment. Arguably, this group represents a profile similar to what is described in much of the literature on the DS phenotype [3]. The next largest group of participants represented more than a third of the sample (35%) and was termed the “cognitive” class. These participants were distinguished by the lowest scores on adaptive behavior and performance-based measures of cognition (IQ and visuospatial abilities) coupled with high ASD symptomatology, executive functioning impairments, and relatively low maladaptive behaviors. The final and smallest group (17%) was termed the “behavioral” class due to high rates of maladaptive behavior, particularly for conduct problems and anxiety, along with high ASD symptomatology and high levels of executive functioning impairment. However, their performance-based cognitive scores (IQ and visuospatial abilities) were similar to that of the “normative” class. Interestingly, the adaptive behavior scores of the “behavioral class” fell in between the other two classes.

The nuanced profiles identified by the “cognitive” and “behavioral” classes not only exemplify the inter-individual variability observed in the DS phenotype but also the intra-individual variability among developmental constructs. That is, it has been difficult to understand the relations among cognition, adaptive and maladaptive behavior, and ASD symptomatology at the group level in DS. However, by using LPA, we were able to observe differential patterns across these constructs within the different classes. For example, as expected, adaptive behavior was strongest in the participants with higher cognition, as measured by IQ and visuospatial abilities, and fewer maladaptive behaviors (“normative” class). Conversely, adaptive behavior was weakest in the participants with lower cognition (“cognitive” class). Interestingly, the “behavioral” class demonstrated a group of individuals with DS with adaptive behavior that fell in line with the full sample’s mean, despite displaying the highest rates of maladaptive behaviors and executive functioning impairments. This is somewhat surprising because maladaptive behaviors and ASD symptomatology tend to be associated with greater difficulties in adaptive behavior [81]. Our results suggest that for individuals with DS, adaptive behavior may be less dependent on these constructs. It is possible that the “behavioral” class captured some children with DS and comorbid developmental behavioral disorder who display high rates of externalizing maladaptive behaviors with a more inconsistent presentation of adaptive behavior (e.g., maladaptive behaviors, such as refusal, that only sometimes impede adaptive function). It is also possible that executive functioning impairments are contributing to this discrepancy, impeding certain aspects of behavior more than others or in certain settings (e.g., school vs. home). Additional research is needed to examine this subgroup of individuals with DS in more depth.

Two of the groups (“cognitive” and “behavioral” classes) demonstrated similar levels of ASD symptomatology despite showing divergence in maladaptive behavior and cognition. This is a particularly noteworthy finding because it suggests that there is more than one phenotype associated with ASD risk in individuals with DS. That is, individuals with DS who have relatively low IQ and visuospatial abilities or who have high rates of maladaptive behavior are at greater risk for ASD. This is consistent with the broader literature on DS showing that ASD symptoms are associated with lower IQ and maladaptive behaviors [61, 62]. However, in the current study maladaptive behavior and performance-based measures of cognition appear to be separately associated with ASD symptomology. These data also suggest that it is possible to disentangle ASD symptomatology from other maladaptive behaviors. Moving forward, this information will be particularly helpful in the search for biomarkers associated with the various pathways to ASD in this population. Future research should also consider the role of executive functioning difficulties, as relatively high rates of impairment were present in both the “cognitive” and “behavioral” classes, with highest rates in the “behavioral” class. Further, although executive functioning is part of cognition, it appears that there are nuances within the skills that make up this large construct in DS that differentially impact functioning.

Importantly, the current study’s findings have identified three distinct subgroups of individuals with DS that are likely also clinically meaningful. When developing interventions for individuals with DS, researchers should consider potential subgroups for which more targeted interventions may work best, rather than determining efficacy (or lack thereof) among a large heterogeneous group. These data also demonstrate the national movement towards precision medicine as applied to DS. Take, as an example, interventions for maladaptive behavior in individuals with DS. Although developmentally-based educational intervention and behavioral management principles still apply, thoughtful consideration regarding when to introduce certain types of intervention and in what sequence is also important. In addition, consideration and treatment of underlying physiologic mechanisms that drive hyperactivity/impulsivity, compulsive/perseverative behavior, anxiety/fearfulness, mood irritability, and self-injury are important for these interventions because they can impact learning and adaptive function. Similarly, the physiologic consequences of untreated medical comorbidities such as sleep disturbance/deprivation, obstructive sleep apnea, and thyroid imbalance can interfere with executive functioning, self-regulation, learning, and development despite ongoing educational and behavioral interventions. Further investigation into their role in shaping cognitive-behavioral subtypes will be critical to developing more personalized and precise treatments. Long term, taking a precision medicine approach optimizes neuromaturation and developmental outcomes for individuals with DS.

Variability in cognition and behavior was not driven by many of the demographic variables in our sample. The only significant differences observed were that lower cognitive functioning (IQ and visuospatial abilities) was associated with being male and lower household income. Household income has been historically linked to child cognition [82], and male sex is a general risk factor for both ASD and intellectual disability [83]. Therefore, future work should also consider genetic and environmental differences, as well as the interaction between the two, to explain the phenotypic profiles observed in this study. Additional child (e.g., perinatal exposures), parental (e.g., stress), and family-related (e.g., broader ASD phenotype in siblings) variables should be considered as well.

The field of DS research has expressed the need for larger sample sizes that can capture the variability associated with the phenotype and that can begin to address larger questions regarding the mechanisms underlying the various expressions of the phenotype [5]. A major strength of this study is that we achieved a large sample size by gathering data across a network of sites. This, in turn, allowed us to use modern statistical methods appropriate for larger samples to address the variability among individuals with DS.

Despite the strengths of our large sample size, we were still limited by a convenience sample. For example, although our sample is in line with the US census data in terms of the percentage of participants who were White [84], it differs in the distribution of minorities. We had a higher percentage of participants who were Asian and a lower percentage who were Black and Hispanic. Still, our sample was more ethnically diverse than many samples of people with DS, likely due to the larger sample size and multiple sites. Nonetheless, further exploration of how these profiles appear in more diverse samples is needed. An additional limitation related to sampling is that some of our measures were performance-based, requiring families to participate in person. Thus, individuals and families unable to participate in demanding assessment batteries were excluded. As a result, our findings may underestimate the prevalence of those with more behavioral and cognitive difficulties. These individuals may present with a different profile than what was represented in the three classes observed in the current study. For example, they may constitute a fourth class for which we did not have an adequate number of participants to adopt from our data.

We were limited also by the number of observations for which we had an adequate sample size, thus limiting the measures included. Some constructs were not represented, most notably language. Because expressive language is an area of particular weakness in the DS phenotype and is often a target of interventions, it is critical to include moving forward. Techniques such as expressive language sampling will be particularly fruitful due to the ability to circumvent the issue of floor effects associated with standardized norm-referenced tests while preserving psychometric quality [85, 86]. To this point, other measures of cognition in future research should be mindful of the assessment form and type of score used to minimize floor effects [87]. Finally, due to the wide age range of participants, we used age-corrected scores in analyses. Although this approach minimized confounds, it potentially limits our understanding of age differences across the subgroups. Future research should consider whether the patterns observed across subgroups of individuals with DS change over time, with age or across different developmental periods.

## Conclusions

As Karmiloff-Smith et al. emphasized, we must consider individual differences at multiple levels (e.g., genetic, epigenetic, neural, cognitive, behavioral, and environmental [5]). The current study took an important first step by identifying within-syndrome patterns of cognition and behavior. Moving forward, the identification of subgroups within the phenotype associated with DS will be a catalyst for biomarker studies aimed at identifying mechanisms underlying the variability observed in the phenotype. To achieve the ultimate goal of more targeted interventions, we need to better understand the pleiotropic effects observed among individuals with DS who share a common genetic etiology.

## Supplementary Information

**Additional file 1 : Figure S1.** Latent Profile Analysis 4-Class Model**.** *Note.* All values are z-score transformed (*M* = 0; *SD* = 1). Higher scores on the NCBRF, SCQ, and BRIEF indicate greater impairment; higher scores on all other measures reflect less impairment.

## Data Availability

The datasets used and/or analyzed during the current study are available from the corresponding author upon reasonable request.

## Abbreviations

DS: Down syndrome
LPA: Latent profile analysis
ASD: Autism spectrum disorder
KBIT-2: Kaufman Brief Intelligence Test, 2nd edition
CANTAB: Cambridge Neuropsychological Test Automated Battery
BRIEF: Behavioral Rating Inventory of Executive Function
SIB-R: Scales of Independent Behavior-Revised, Short Form
NCBRF: Nisonger Child Behavior Rating Form
SCQ: Social Communication Questionnaire
AIC: Akaike information criterion
SS-BIC: Sample size-adjusted Bayesian information criterion
VLMR: Vuong-Lo-Mendell-Rubin
BLRT: Bootstrapped likelihood ratio
M(CP): Mean class probability
FIML: Full information maximum likelihood estimation
