# Supplementary material for: Capturing cognitive and behavioral variability among individuals with Down syndrome: a latent profile analysis
Source: J Neurodev Disord. 2021 Apr 19;13:16. doi: 10.1186/s11689-021-09365-2 (PMC8056665; doi:10.1186/s11689-021-09365-2)
Supplement: Supplementary file 1 — Additional file 1 : Figure S1. Latent Profile Analysis 4-Class Model. Note. All values are z-score transformed (M = 0; SD = 1). Higher scores on the NCBRF, SCQ, and BRIEF indicate greater impairment; higher scores on all other measures reflect less impairment. [file 11689_2021_9365_MOESM1_ESM.docx]

**SUPPLEMENTAL INFORMATION**

**Figure 1**

*Latent Profile Analysis 4-Class Model*

*Note.* All values are z-score transformed (*M* = 0; *SD* = 1). Higher scores on the NCBRF, SCQ, and BRIEF indicate greater impairment; higher scores on all other measures reflect less impairment.
